# The Role of Frequent Screening or Diagnostic Testing of Serum Cryptococcal Antigen in Liver Transplant Recipients: A Descriptive Epidemiology

**DOI:** 10.1093/ofid/ofae255

**Published:** 2024-05-17

**Authors:** Toshiki Miwa, Koh Okamoto, Kazuhiko Ikeuchi, Shinya Yamamoto, Shu Okugawa, Akihiko Ichida, Nobuhisa Akamatsu, Kiyoshi Hasegawa, Takeya Tsutsumi

**Affiliations:** Department of Infectious Diseases, The University of Tokyo Hospital, Tokyo, Japan; Department of Infectious Diseases, The University of Tokyo Hospital, Tokyo, Japan; Department of Infectious Diseases, Graduate School of Medical and Dental Sciences, Tokyo Medical and Dental University, Tokyo, Japan; Department of Infectious Diseases, The University of Tokyo Hospital, Tokyo, Japan; Department of Infectious Diseases, The University of Tokyo Hospital, Tokyo, Japan; Department of Infectious Diseases, The University of Tokyo Hospital, Tokyo, Japan; Artificial Organ and Transplantation Surgery Division, Department of Surgery, Graduate School of Medicine, The University of Tokyo, Tokyo, Japan; Artificial Organ and Transplantation Surgery Division, Department of Surgery, Graduate School of Medicine, The University of Tokyo, Tokyo, Japan; Artificial Organ and Transplantation Surgery Division, Department of Surgery, Graduate School of Medicine, The University of Tokyo, Tokyo, Japan; Department of Infectious Diseases, The University of Tokyo Hospital, Tokyo, Japan

**Keywords:** cryptococcosis, cryptococcal antigen, asymptomatic cryptococcal antigenemia, diagnostic stewardship, liver transplant

## Abstract

**Background:**

Cryptococcosis is a notable infectious complication of liver transplantation. Currently, there is no recommendation for screening serum cryptococcal antigen (CrAg) levels in solid organ transplant recipients. We aimed to explore the role of serum CrAg in liver transplant recipients at an institution where posttransplant serum CrAg has been widely tested.

**Methods:**

This retrospective study was conducted at a tertiary care center in Japan. All liver transplant recipients with serum CrAg measured either for screening or for diagnostic testing at least once after transplantation between April 2005 and March 2022 were included. For participants with either a positive CrAg test result or positive culture for *Cryptococcus*, we manually reviewed clinical manifestations, management, and prognosis from the medical records.

**Results:**

During the study period, 12 885 serum CrAg tests (median, 16 tests per patient) were performed in 468 liver transplant recipients. The 1-year posttransplant incidence of positive serum CrAg test results and culture-proven cryptococcosis was 1.9% (9/468) and 0.6% (3/468), respectively. No patient with persistently negative serum CrAg test results showed growth of *Cryptococcus* in culture. Four patients had clinical manifestations consistent with cryptococcosis, of whom 2 (50.0%) started antifungal therapy promptly based on a positive serum CrAg test result. In contrast, 5 patients had no clinical manifestations. Three of the 5 (60.0%) patients did not receive antifungal therapy and remained free of clinical manifestations.

**Conclusions:**

Serum CrAg test was more sensitive than culture among liver transplant recipients and prompted early diagnosis and antifungal therapy in symptomatic patients. However, serial screening of serum CrAg in asymptomatic patients may be of little value, with the potential for false-positive results.

## Teaser

Serum cryptococcal antigen tests potentially prompt the diagnosis and antifungal therapy in liver transplant recipients with compatible clinical manifestations. However, frequent screening in all liver transplant recipients may be of little value given the low prevalence of cryptococcosis.

Cryptococcosis is the third most common invasive fungal infection after candidiasis and aspergillosis in solid organ transplant (SOT) recipients [1]. Cryptococcosis in patients without human immunodeficiency virus (HIV) infection, including SOT recipients, has distinct characteristics compared with that in patients with advanced HIV infection. Diagnosis is often delayed in patients without HIV infection owing to atypical presentation. Although the reported mortality is highly variable, perhaps due to the difference in healthcare resources including drug availability [2], the mortality among patients without HIV infection remains high, which underscores the importance of early differential diagnosis and prompt management [3–7].

Cryptococcosis in liver transplant recipients is of particular concern because end-stage liver failure is a well-known risk factor for cryptococcosis [8]. Cryptococcosis in liver transplant recipients is rare; the incidence of culture-proven cryptococcosis was 0.3%–0.6% in several single-center studies [9–13], and the 12-month posttransplant cumulative incidence was 0.4% in a large cohort study in the United States [14]. Nevertheless, compared with other SOTs, liver transplantation has been associated with the development of disseminated infection [15], making cryptococcosis a notable morbidity among liver transplant recipients.

Serum cryptococcal antigen (CrAg) tests can be used to detect the early phase of cryptococcosis in SOT recipients [16, 17]. The role of CrAg in the diagnosis of cryptococcosis has been extensively investigated among patients with advanced HIV infection, including the benefit of using serum CrAg as a screening test regardless of symptoms [18]. However, the findings of these studies may not apply to SOT recipients because the prevalence of cryptococcal antigenemia among patients with advanced HIV infection (CD4 count <100 cells/μL) was estimated to be 6.0% globally with significant geographical variation [18–20], which is significantly higher than that among SOT recipients. Although a quarterly serum CrAg screening in SOT recipients was predicted to be “at least cost neutral” [21], currently, there is no recommendation for frequent screening of serum CrAg levels in SOT recipients because of the lack of studies based on real-world data [22].

This study aimed to explore the role and effectiveness of serum CrAg testing in liver transplant recipients at a center where posttransplantation serum CrAg has been widely tested either for screening or for diagnostic testing. We also aimed to assess whether a positive serum CrAg test facilitates the diagnosis and treatment of cryptococcosis and whether there is a potential overdiagnosis owing to false-positive serum CrAg test results in this setting.

## METHODS

### Study Design and Setting

This retrospective, single-center study was conducted at the University of Tokyo Hospital, a 1226-bed tertiary care center located in Tokyo, Japan. The University of Tokyo Hospital is one of the major liver transplantation centers in Japan, and 798 living donor liver transplantations (LDLTs) and 64 deceased donor liver transplantations (DDLTs) were performed between January 1996 and December 2022. At our institution, liver transplant recipients received a 5-day course of ampicillin-sulbactam and cefotaxime and a 7-day course of micafungin perioperatively as prophylactic antimicrobials. In addition, according to the institutional protocol, the serum CrAg qualitative test was ordered preoperatively at the time of transplant registry and on admission, twice a week postoperatively until discharge, and when patients were hospitalized again, regardless of symptoms. This frequent serum CrAg screening strategy is based on our experience that serum CrAg tests may be effective for detecting cryptococcosis in liver transplant recipients at the early stage [16]. Additional CrAg tests, either qualitative or semiqualitative, were performed at the discretion of surgeons in both ambulatory and inpatient settings. Because the study institution outsourced the CrAg tests (latex agglutination assays, qualitative test: SRL, Inc, Japan; semiquantitative test: LSI Medical Co, Japan; both assays used Serodirect [Eiken Chemical Co, Ltd, Japan]), there was a turnaround time of approximately 3 days. The results of the CrAg tests were not routinely reviewed by the Department of Infectious Diseases; however, infectious disease consultation services were available upon request from transplant surgeons.

Per the institutional protocol, the initial immunosuppressive regimen consisted of high-dose corticosteroids and calcineurin inhibitors (CNIs), mainly tacrolimus. Maintenance immunosuppressive therapy included corticosteroids and CNIs. We add on mycophenolate mofetil (MMF) to reduce the dose of CNIs in recipients with impaired renal function. Exceptions were the patients receiving ABO-incompatible liver transplantation, in whom rituximab was administered to prevent antibody-mediated rejection [23] and a triple-drug regimen consisting of corticosteroids, CNIs, and MMF was used as a maintenance therapy.

### Participants

This study included patients who underwent liver transplantation at our institution at age ≥18 years between April 2005 and March 2022. Patients who did not undergo postoperative serum CrAg testing during the study period were excluded.

### Data Collection and Analyses

We retrospectively collected the results of all serum CrAg tests in all the study participants through electronic health records. To calculate the incidence of positive CrAg test results, we followed up each patient until the end of the study period, death, loss to follow-up, or initial positivity for serum CrAg, whichever occurred first. In addition, cerebrospinal fluid (CSF) positive for CrAg and cultures of *Cryptococcus* species, including blood and CSF specimens after transplantation, were obtained from the database of the microbiology laboratory. For patients with either serum CrAg positivity, CSF CrAg positivity, or a positive culture for *Cryptococcus* at least once, we manually reviewed the medical records to obtain demographic data, clinical assessment, clinical course, and the presence or absence of antifungal therapy active against *Cryptococcus* in vitro. Disseminated cryptococcosis included fungemia, meningitis, high titer of CrAg (≥1:160) predicting meningitis, and an infection with at least 2 positive nonadjacent site cultures [24–26]. Serum CrAg test was interpreted as true positive if a study participant had a positive culture for *Cryptococcus* from any site or clinical manifestations consistent with cryptococcosis, including pneumonia, bloodstream infection, meningitis, or sepsis [22]. Otherwise, serum CrAg was defined as isolated antigenemia.

### Patient Consent Statement

This study was approved by the Institutional Review Board of the University of Tokyo Hospital (approval number 2023046NI). Given the retrospective nature of the study, the requirement for patient consent was waived in accordance with the Declaration of Helsinki. This study was performed in accordance with the Strengthening the Reporting of Observational Studies in Epidemiology (STROBE) guidelines.

## RESULTS

### Effectiveness of Frequent Testing of Serum CrAg Levels in Liver Transplant Recipients

**Figure 1.**
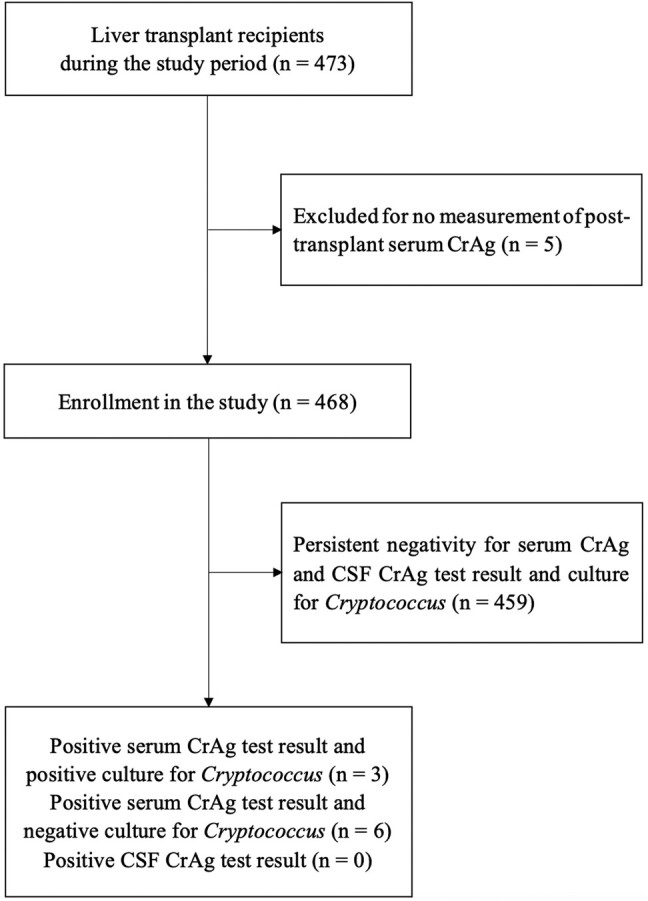
Flow diagram of participant selection. Abbreviations: CrAg, cryptococcal antigen; CSF, cerebrospinal fluid.

During the study period, our institution performed 480 liver transplant surgeries (427 LDLT and 53 DDLT) in 473 individuals, including 7 recipients who underwent a second liver transplantation. Pretransplant serum CrAg tests were performed on 471 recipients (471/473 [99.6%]) (424 LDLT and 47 DDLT). Of the 473 recipients, 3 recipients had no posttransplant serum CrAg test results for unknown reasons, and 2 recipients whose liver transplantation was performed in March 2022 did not undergo posttransplant serum CrAg tests by the end of the study period. None of these 5 recipients had experienced cryptococcosis subsequently. Finally, 468 individuals (468/473 [98.9%]) (422 LDLT and 46 DDLT) who underwent serum CrAg tests at least once after liver transplantation were left for further analysis (Figure 1).

**Figure 2.**
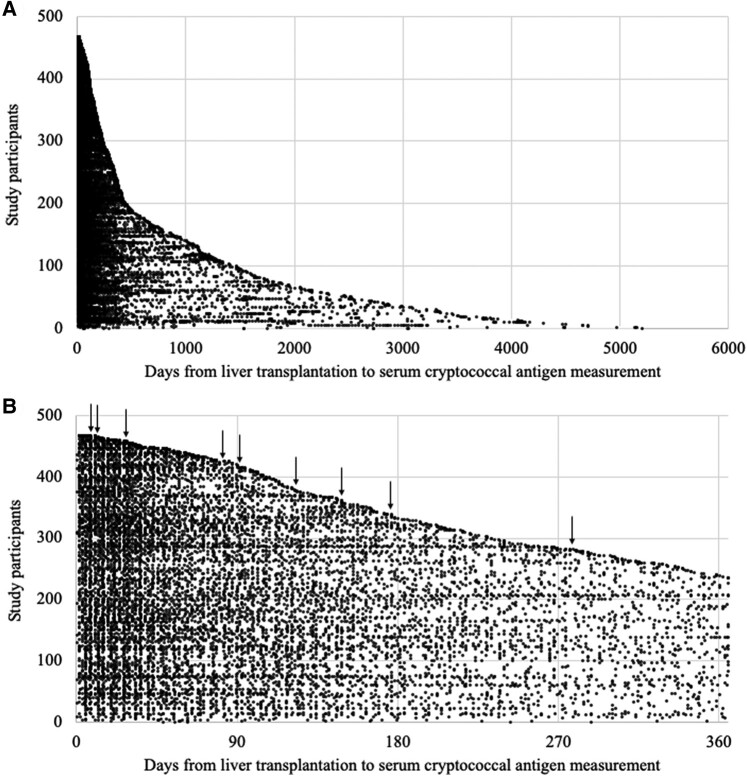
Distribution of the timing of measuring serum cryptococcal antigen (CrAg) following liver transplantation. The timing of serum cryptococcal antigen measurement in each study participant is plotted horizontally. In total, 468 patients are sorted vertically by the length of follow-up duration. Distribution of the timing was depicted throughout the follow-up period (*A*) and within 365 days from liver transplantation (*B*). Arrows indicate positive serum CrAg.

The median age of patients who underwent at least 1 posttransplant serum CrAg test was 54 years (interquartile range [IQR], 44–59 years). Male recipients accounted for 52.8% (247/468) of the patients. Among 468 study participants, 2 were HIV positive (0.4%), while others were all HIV negative. Viral hepatitis was the primary indication for liver transplantation (163/468 [34.8%]), followed by autoimmune liver disease (128/468 [27.4%]). The median observation duration was 44 months (interquartile range [IQR], 14–124 months). For this population, 12 885 serum samples (median, 16 [IQR, 8–29] per patient) were submitted for CrAg testing during this period, either as a screening test for asymptomatic patients or as a diagnostic test for symptomatic patients (Figure 2).

Pretransplant screening for serum CrAg led to the diagnosis of cryptococcal pneumonia in 1 candidate for liver transplant during the study period, resulting in the suspension of surgery. For the study participants, serum CrAg tests were consistently performed at a median of 19 days before transplantation (IQR, 7–28 days), and all were negative. In contrast, posttransplant serum CrAg was positive in 9 patients (9/468 [1.9%]), all of whom were HIV negative. There was a tendency that male patients were more likely to have positive serum CrAg tests than female patients (7/240 [2.9%] vs 2/219 [0.8%]). All the posttransplant serum CrAg positivity occurred within 1 year posttransplantation. The incidence rates at 0–3, 3–6, and 6–12 months posttransplant were 0.098 per 1000 patient-days (4 cases/40 660 patient-days), 0.106 per 1000 patient-days (4 cases/37 736 patient-days), and 0.014 per 1000 patient-days (1 case/70 647 patient-days), respectively. Three of the 9 patients (33.3%) also had positive cultures for *Cryptococcus* (blood cultures in 2 patients and pleural effusion and ascites cultures in the remaining patient), in addition to positive serum CrAg tests, all of which were identified as *Cryptococcus neoformans*. Of the remaining 6 patients, blood and CSF cultures were assessed in 2 patients and 1 patient, respectively, all of which were negative. The 1-year posttransplant incidence of culture-proven cryptococcosis was 0.6% (3/468) (Table 1). Patients with persistently negative posttransplant serum CrAg test results had no cultures positive for *Cryptococcus* or positive CSF CrAg test results. The overall test fee for serum CrAg tests (1440 Japanese yen or 9.5 US dollars [USD] for each test) was 122 408 USD, indicating that it cost 13 601 USD to identify each positive serum CrAg case.

**Table 1.** Characteristics of Liver Transplant Recipients With Subsequent Serum Cryptococcal Antigen Tests Performed at Least Once

Variables	Overall (N = 468)
Demographics	
Age at liver transplantation, y, median (IQR)	54 (44–59)
Male sex	247 (52.8)
Comorbidities	
HIV-positive recipients	2 (0.4)
Liver transplantation	
Living donor liver transplantation	422 (90.2)
Deceased donor liver transplantation	46 (9.8)
Underlying liver disease	
Viral hepatitis	163 (34.8)
Autoimmune liver diseases^a^	128 (27.4)
Alcoholic hepatitis	51 (10.9)
Nonalcoholic fatty liver disease	43 (9.2)
Hepatocellular carcinoma	97 (20.7)
Cryptococcal tests	
No. of serum antigen tests per patient, median (IQR)	16 (8–29)
Interval between liver transplantation and the day of test, d, median (IQR)	96 (34–243)
Positive serum antigen test and positive culture	3 (0.6)
Positive serum antigen test and negative culture	6 (1.3)
Negative serum antigen test and positive culture	0

Data are presented as No. (%) unless otherwise indicated.

Abbreviations: HIV, human immunodeficiency virus; IQR, interquartile range.

^a^Autoimmune liver diseases include autoimmune hepatitis, primary biliary cholangitis, and primary sclerosing cholangitis.

### Characteristics of Patients With Positive Serum CrAg Test or Culture for *Cryptococcus*

Tables 2 and 3 show the characteristics of 9 patients with positive serum CrAg test results after liver transplantation. Seven of the 9 patients (77.8%) were male. Eight patients (88.9%) had positive serum CrAg test results within 180 days of liver transplantation, and 3 patients (33.3%) had positive serum CrAg test results within 30 days of transplantation.

**Table 2.** Baseline Characteristics of Patients With Positive Serum Cryptococcal Antigen Tests Following Liver Transplantation

Patient No.	Age/Sex^a^	Indication for Liver Transplant	Donor Type	Initial Immunosuppressive Regimen	Maintenance Immunosuppressive Therapy at the Time of Positive CrAg	Other Immunosuppressing Factors	History of Acute Rejection Within 90 Days of Positive CrAg
1	60/M	PBC	Living donor	mPSL, Tac	mPSL 14 mg, Tac 7 mg	None	Yes
2	49/M	C-LC	Living donor	mPSL, Tac	mPSL 6 mg, Tac 3 mg	Immunosuppressive therapy for UC	No
3	65/F	C-LC and HCC	Living donor	mPSL, Tac	mPSL 2 mg, Tac 3 mg	None	No
4	66/M	ALC-LC	Living donor	mPSL, Tac	mPSL 8 mg, CsA 10 mg	None	No
5	45/M	ALF	Living donor	mPSL, Tac	mPSL 8 mg, Tac 4.5 mg	None	No
6	57/F	PBC	Living donor	mPSL, Tac	mPSL 6 mg, Tac 10 mg, MMF 1500 mg	None	No
7	56/M	PSC	Living donor	mPSL, Tac	mPSL 36 mg, Tac 6 mg	None	No
8	41/M	ALC-LC	Living donor	mPSL, Tac	mPSL 60 mg, CsA 100 mg	None	Yes
9	35/M	IPH	Living donor	mPSL, Tac	mPSL 80 mg, Tac 6 mg	Immunosuppressive therapy for ARDS	No

Abbreviations: ALF, acute liver failure; ALC-LC, alcoholic liver cirrhosis; ARDS, acute respiratory distress syndrome; C-LC, liver cirrhosis due to chronic hepatitis C infection; CrAg, cryptococcal antigen; CsA, cyclosporine; F, female; HCC, hepatocellular carcinoma; IPH, idiopathic portal hypertension; M, male; MMF, mycophenolate mofetil; mPSL, methylprednisolone; PBC, primary biliary cholangitis; PSC, primary sclerosing cholangitis; Tac, tacrolimus; UC, ulcerative colitis.

^a^Patient's age at the time of liver transplantation.

**Table 3.** Microbiological Characteristics, Management, and Prognosis of Patients With Positive Serum Cryptococcal Antigen Tests Following Liver Transplantation

Patient No.	Time From Transplant to the Last Negative Test, d	Time From Transplant to the First Positive Test, d	No. of Tests^a^	Positive Culture for *Cryptococcus*	Presentations	Clinical Diagnosis^b^	Antifungal Therapy	Persistence of Positive Serum CrAg, d^c^	1-y Survival^d^	Interpretation of Serum CrAg
1	79	82	4	Negative	No symptoms	NA	FLCZ	1	Death^e^	Isolated antigenemia
2	62	149	6	Negative	Fever	Cholangitis	No	1	Survival	Isolated antigenemia
3	244	279	21	Negative	No symptoms	NA	No	1	Survival	Isolated antigenemia
4	84	91	15	Blood	Fever	Bloodstream infection	FLCZ	65	Survival	Disseminated infection
5	115	122	5	Negative	No symptoms with lung opacities	Pneumonia	FLCZ	322	Survival	Pneumonia
6	141	176	22	Negative	No symptoms	NA	No	29	Survival	Isolated antigenemia
7	7	10	4	Pleural effusion, ascites	Somnolence	Sepsis	L-AmB followed by FLCZ	393	Survival	Disseminated infection
8	10	13	7	Negative	No symptoms	NA	L-AmB and 5-FC followed by FLCZ	95	Survival	Isolated antigenemia
9	24	28	7	Blood	Respiratory failure	Sepsis	L-AmB and 5-FC followed by FLCZ	Persistent positive to death	Death^f^	Disseminated infection

Abbreviations: 5-FC, flucytosine; CrAg, cryptococcal antigen; FLCZ, fluconazole; L-AmB, liposomal amphotericin B; NA, not applicable.

^a^The number of serum cryptococcal antigen tests performed until the positive result occurred.

^b^Clinical diagnosis at the time of positive serum CrAg test.

^c^Subsequent CrAg tests were negative in all patients.

^d^One-year survival or mortality from the first positive serum CrAg result.

^e^The patient died of uncontrolled bacterial liver abscess 3 months later.

^f^The patient died of refractory respiratory failure following *Stenotrophomonas maltophilia* bacteremic pneumonia and disseminated cryptococcosis 2 months later.

Four patients had a clinical diagnosis of pneumonia, bloodstream infection, or sepsis; thus, their serum CrAg positivity was interpreted as a true-positive result. Three of the 4 patients had cultures positive for *Cryptococcus* (patients 4, 7, and 9). Among these 4 patients, positive serum CrAg test results led to prompt therapy in 2 patients before culture results were available (patients 5 and 9) (2/4 [50.0%]). Patients 4 and 5 received fluconazole monotherapy, patient 7 received liposomal amphotericin B monotherapy followed by fluconazole monotherapy, and patient 9 received a combination of liposomal amphotericin B and flucytosine followed by fluconazole monotherapy. Among the 4 patients, the shortest duration of serum CrAg positivity was 65 days. Patient 9 died of uncontrolled disseminated cryptococcosis while receiving this regimen.

The other 5 patients with positive serum CrAg test results did not have clinical manifestations suggestive of cryptococcosis. Three of the 5 patients (60.0%) did not receive antifungal therapy. Semiquantitative serum CrAg was up to 1:128 in patient 8 but was not measured in the other 4 patients. The duration of positive serum CrAg tests varied from 1 day (patients 1, 2, and 3) to 95 days (patient 8), and we confirmed that subsequent CrAg tests were negative in all patients. Patient 1 received 4 weeks of fluconazole monotherapy, and patient 8 received 2 weeks of liposomal amphotericin B and flucytosine followed by about 8 months of fluconazole therapy, respectively. Patients 2 and 3 and patient 6 received no antifungal therapy. None of these 5 patients had culture-proven cryptococcosis. Four patients survived for more than a year, whereas 1 patient died from an uncontrolled bacterial liver abscess within a year of serum CrAg positivity.

## DISCUSSION

The present study showed that the 1-year incidences of positive serum CrAg test results and culture-proven cryptococcosis in liver transplant recipients under frequent testing of serum CrAg were 1.9% and 0.6%, respectively. A median of 16 serum CrAg tests were performed in 468 patients and identified 9 patients with positive serum CrAg test results at the study institution. In half of the patients with clinical manifestations consistent with cryptococcosis, positive serum CrAg test results led to early recognition of cryptococcosis and initiation of antifungal therapy before the culture results were available. However, in other patients, no clinically apparent cryptococcosis subsequently developed, and their prognosis was generally favorable even without antifungal therapy. These findings suggest that serum CrAg tests should be used for diagnostic testing in patients with compatible symptoms, rather than for serial screening of all posttransplant patients.

The utility of CrAg screening has been extensively studied in treatment-naive patients with HIV infection. A previous study suggested that CrAg was detected in the blood before clinical symptoms developed in this population [27], and another clinical trial supported the strategy of screening and preemptive therapy based on CrAg tests [28]. However, there are scant data on such a strategy in SOT recipients. In our cohort, no cases of cryptococcosis were overlooked because of negative CrAg results. However, 12 885 CrAg tests were required to identify 9 patients with serum CrAg positivity. Given the cost and workload in the laboratory, serial screening of CrAg in all liver transplant recipients may not be an efficient approach, especially beyond a year posttransplantation. The difference in the role of serial CrAg screening between SOT recipients and patients with advanced HIV infection may be explained by the difference in the epidemiology of cryptococcosis. First, the incidence of cryptococcosis in SOT recipients is lower than that in patients with advanced HIV infection [1, 19]. Second, the timing of disease onset after transplantation varies among SOT recipients. While the risk of cryptococcosis in patients with HIV infection declines sharply once antiretroviral agents are started [29], the timing of cryptococcosis in SOT recipients varies from very early onset (within 30 days of transplantation) to late onset (beyond a year of transplantation) [30].

Our findings suggest that targeted CrAg screening may be a better approach than universal CrAg screening in liver transplant recipients. Some risk factors may be useful for risk stratification. The time from liver transplant is likely a key factor. Consistent with previous reports [30, 31], our study indicated that patients within 6 months of liver transplant were at the highest risk for cryptococcosis. Serum CrAg screening strategy may become more efficient if it only targets this population. Male sex was proposed as a risk factor for the development of cryptococcosis by a previous study [32]. A screening strategy might be more useful in an area with higher prevalence because there is a considerable regional variation in the prevalence of cryptococcosis [19]. Of note, disseminated cryptococcosis is a nationally notifiable disease in Japan, and its incidence among the entire population in 2020 was 1.2 per million, although data on HIV status are unavailable [33]. Furthermore, the intensity of immunosuppression may also need to be taken into consideration [22]. However, we did not identify notable characteristics in the immunosuppressive regimen or a recent history of rejection in patients with positive serum CrAg tests, possibly due to a small sample size, nor obtained the data on those whose CrAg tests were persistently negative. Therefore, the evaluation of a targeted CrAg screening approach based on the intensity of immunosuppression warrants further study.

The potential role of fungal biomarkers, such as CrAg, is to facilitate the diagnosis of invasive fungal infection [34]. In the present study, serum CrAg positivity led to the early recognition of cryptococcosis and initiation of antifungal therapy without awaiting culture results in some patients with compatible symptoms. This finding suggests that serum CrAg testing should be encouraged in liver transplant recipients with clinical manifestations consistent with cryptococcosis, including pneumonia, unexplained fever, or sepsis in addition to meningitis.

Serum CrAg testing is considered highly specific for cryptococcosis, and the percentage of false-positive cases has been reported as 0–0.4%; false-positive results have been associated with the presence of rheumatoid factor or other infections, such as trichosporonosis [29]. In patients with advanced HIV infection, cryptococcal antigenemia precedes the development of symptomatic cryptococcosis in the absence of compatible manifestations; therefore, it is treated with antifungal agents [35]. Notably, when cryptococcosis was defined based on positive culture in our study, its incidence was almost comparable to that among liver transplant recipients in previous studies where asymptomatic cryptococcal antigenemia was not included [9–11]. However, the incidence in our study tripled if all positive serum CrAg test results regardless of clinical manifestation were included as a part of the definition. In addition, some culture-negative, CrAg-positive liver transplant recipients did not experience subsequent clinically apparent cryptococcosis and survived for more than a year without antifungal therapy. These findings suggest that isolated cryptococcal antigenemia may include false-positive CrAg results. Therefore, extensive screening of serum CrAg levels in the absence of clinical suspicion of cryptococcosis in liver transplant recipients may lead to unnecessary antifungal treatment and may be of little value.

Interestingly, all patients with clinically apparent cryptococcosis had at least 65-day persistence of positive serum CrAg tests. This finding suggests that a serum CrAg test that is positive only once in asymptomatic patients may be a clue to identifying a false-positive result.

The present study had several limitations. First, given the incomplete documentation, we were unable to ascertain the indications for each serum CrAg test or the rationale for deferring antifungal therapy in patients with positive serum CrAg test results. Second, blood cultures, CSF cultures, CSF CrAg, and semiquantitative evaluation of serum CrAg, all of which may help distinguish subclinical cryptococcosis from false-positive serum CrAg test results in SOT recipients [36], were inconsistently performed in this study. Third, we did not collect pathological data; therefore, we may have overlooked cryptococcosis diagnosed only using tissue biopsy. However, considering the relatively sensitive nature of CrAg tests [29] and the consistency in the incidence of cryptococcosis in the present and previous studies, it is unlikely that we missed cases. Fourth, our results may not be applicable to healthcare settings that use the CrAg lateral flow assay, which is slightly more sensitive than the CrAg latex agglutination assay [34].

In conclusion, serum CrAg tests are more sensitive than culture tests in liver transplant recipients and may have the potential for prompt diagnosis and antifungal therapy in a compatible clinical context. However, given the low prevalence of cryptococcosis, frequent screening of serum CrAg in all liver transplant recipients may include false-positive results and may be of little value, especially when performed beyond 1 year posttransplantation.

## Notes

***Acknowledgments***. We would like to thank Editage (www.editage.jp) for English-language editing.

***Author contributions***. T. M., K. O., N. A., and K. H. designed the study. T. M. and K. O. obtained data. T. M. and K. O. analyzed and interpreted the data. T. M. drafted the manuscript. N. A., K. H., and T. T. supervised the study. All authors critically reviewed the manuscript and approved the submitted version.

***Data availability*.** Data are not publicly available.

***Financial support***. This work was supported in part by the Japan Society for the Promotion of Science KAKENHI (grant number JP 23K16252 to K. O.).
